# Ownership in Name, But not Necessarily in Action: Comment on "It’s About the Idea Hitting the Bull’s Eye": How Aid Effectiveness Can Catalyse the Scale-up of Health Innovations"

**DOI:** 10.15171/ijhpm.2018.72

**Published:** 2018-08-06

**Authors:** Melisa Martinez-Alvarez

**Affiliations:** MRC Unit The Gambia, London School of Hygiene and Tropical Medicine, London, UK.

**Keywords:** Scalability, Scale-up, Ownership, Aid Effectiveness

## Abstract

A recently-published paper by Wickremasinghe et al assesses the scalability of pilot projects in three countries using the aid effectiveness agenda as an analytical framework. The authors report uneven progress and recommend applying aid effectiveness principles to improve the scalability of projects. This commentary focuses on one key principle of aid effectiveness – country ownership; it describes difficulties in defining and achieving it, and provides practical steps donors and recipient governments can take to move forward towards country ownership.

## Introduction

A recently-published paper by Wickremasinghe et al analyses the scalability of three maternal health programs in three different countries using the aid effectiveness agenda as an analytical framework to explore constraints and facilitating factors to programme scalability.^1^ This is a welcome, novel and practical use of the aid effectiveness agenda. The results show that the different aid effectiveness principles were adopted to different degrees in the three settings, and that this had an impact on programme scale-up. The authors therefore conclude that donors, implementers and recipient governments all need to adhere to aid effectiveness principles to increase the scalability of interventions. The article makes particular emphasis on the principle of country ownership, which the authors describe as facilitating donor alignment and harmonisation, being essential to scale up interventions and to guarantee programme sustainability beyond the support of external partners.^1^ Country ownership has been a principle of all declarations prior and subsequent to the Paris Declaration. In the Accra Agenda for Action, which superseded the Paris Declaration, it was considered the “first priority.”

This commentary discusses that despite the importance of country ownership, it is the hardest principle to attain, and one that is often only referred to in a tokenistic manner. Donors and recipient governments need to make meaningful changes to their approach to managing external and domestic funds in the health sector in order to achieve country ownership, which is particularly relevant in light of current demands for increased domestic spending on health.^2^

## What Is Ownership?

Despite the importance of country ownership, it is a concept that is particularly difficult to define. The paper by Wickremasinghe et al adopts the Paris Declaration’s definition, that recipient countries develop their own development strategies, behind which all donors align.^1^ However, this definition is very broad, and difficult to apply and assess in practice. Studies have shown that the principle of country ownership is interpreted differently by country-level and global stakeholders, and from the texts of aid effectiveness declarations.^3,4^ In particular, it is difficult to assess in practice *whose* ownership should be considered, and *how* ownership should be exercised.^3^ Many studies (Wickremasinghe et al included), equate ‘country’ ownership with ‘government’ ownership. However, country ownership could be interpreted as meaning ownership by the citizens of the recipient country, which may not be fully represented by government priorities, and may also be represented by civil society.

Even taking country to mean government ownership, academics and practitioners are still faced with the problem of how to assess whether a government has ownership. The Paris Declaration’s assessment of ownership was limited to counting whether countries had a development strategy in place. What this has meant in reality in countries like Tanzania and Malawi, is that to meet the principle of ownership the government held separate budgetary meetings with donors, but then still made their decisions behind closed doors; the donors could report on these meetings as meeting ownership, but this created a whole parallel system of accounting for resources which was not government-led and did not reflect the priorities of the recipient government.^3,5^ In all cases (including the Paris Declaration), government leadership is seen as essential to country ownership (also to alignment and harmonisation^1,3,6^).

## How Can Ownership Be Achieved?

Despite difficulties with explicitly defining country ownership, there are steps both donors and recipient countries can take to work towards a principle of the government being in charge of its own development strategies, to which all donors align in a harmonised manner. These measures require changing institutional procedures and mentalities, and are therefore difficult to achieve in practice.

From the donor perspective, government ownership requires donors to trust recipient governments, to relinquish the ability of accounting for results achieved with their specific aid, and to change the metrics used to assess aid effectiveness. These are all challenging in the current context. However, there are three strategies donors can follow to strengthen their efforts towards ownership. First, donors need to relinquish some control of their aid funds, and involve the government at all stages of programme planning and development. They need to trust governments’ capacity to plan and manage resources (a capacity they can themselves strengthen). Wickremasinghe and colleagues acknowledge such trust needs time for donors to engage in a dialogue with the recipient countries, something that donor programmes often lack, but also that donors have become frustrated with governments, something that has been reported in a cross-country study of funding modalities that found that donors were increasingly disbursing aid projects outside of government.^7^ In addition, it is important to consider who should be the implementers of donor projects. Should pilots be conducted by civil society organisations, or within the health system in which they would be implemented if they are scaled up? The latter would not only facilitate country ownership, but it may also strengthen the health system in which the pilot is being implemented, and provide lessons for real-life constraints scaling up the intervention.

Second, there are tensions between achieving the principles of ownership and managing for results - an aid effectiveness principle that Wickremasinghe et al surprisingly did not assess. The pressure to achieve and be able to show results from aid funds often runs contrary to the principle of country ownership, where donors give governments more control of what is done with aid funds, leaving them less able account for the use of funds in a way that will satisfy constituencies. During the 2000s donors adopted sector-wide approaches, in which they committed to working with governments, using horizontal funding modalities (such as basket funds, which allowed donors and governments to jointly decide on development strategies). Sector-wide approaches were seen as vehicles to achieving the aid effectiveness agenda.^3^ Studies have shown, however, that pressures to achieve and show results have contributed to donors abandoning these approaches in favour of vertical projects, sometimes managed by non-government actors.^3,7^ One step towards relieving tensions between ownership and accounting for results would be to have one criterion for evaluation of projects be the extent to which they are aligned within recipient country health systems.

Third, it is essential that donors improve the predictability of their project funding, this not only means undertaking forward planning to help recipient governments plan, but also disburse the committed amounts (something not addressed by Wickremasinghe et al^1^).

From the perspective of recipient governments there are also important steps that need to happen in order to ensure they retain ownership of their development strategies. As the examples in Wickremasinghe et al show, strong government leadership and a willingness to engage with donors, will empower governments to take charge of their development strategies.^1^ In addition, it is as important that recipient governments are also transparent about the way they use funds, both domestic and external, and are also predictable both in making long term plans, and in disbursing committed funds. If donors know what governments plans are for future investments, they may also find it easier to align their support. This approach may lay the foundations for a real partnership between donors and governments (as was the spirit of the Paris Declaration). Rather than donors demanding a joint budgetary exercise, if country governments first come up with their development strategies, and donors then choose which parts of these strategies to support, both parties could set targets for expenditure, for which they would both be held accountable. This strategy has recently been employed by the European Union with the government of The Gambia^8^; time will tell whether a real partnership is developed. Such an approach can also help the scalability and sustainability of pilot projects.

Finally, until recently, discussions on ownership have centred around government ownership of their development priorities, which donors subsequently support. In the current discussions of increasing domestic expenditure on health, particularly for countries in economic transition, it is worth considering whether and how pressure from the international community to influence how governments spend their resources respect the principle of country ownership. In recent years, there have been discussions on the effect of aid on domestic health expenditure. Although the literature is inconclusive on the size and direction of the effect, a case study in Tanzania showed that displacement of aid may be a way of governments of exerting ownership, resulting from a lack of meaningful dialogue.^9^ An approach based on country ownership may be the most productive way to achieve an allocation of resources that ultimately benefits the populations of recipient countries.

## Conclusion

The principle of ownership remains as relevant and important as when it was first introduced in aid effectiveness declarations. Although the international community has maintained the importance of country ownership throughout the different declarations (unlike alignment for example), difficulties in its implementation, broad nature, and tensions with showing and achieving results, have meant that the principle of ownership has remained little more than a box-ticking exercise.

The paper by Wickremasinghe et al provides practical strategies for achieving ownership^1^; such an applied approach is extremely useful for practitioners in the field (both from the donor and from the recipient government perspective). The key may lie in having a dialogue where governments make their priorities known, donors can invest in pilots to test the most effective and cost-effective way to achieve those priorities, which can then be taken on by the government.

## Ethical issues

Not applicable.

## Competing interests

Author declares that she has no competing interests.

## Author’s contribution

MMA is the single author of the paper.
